# Extracellular vesicle-associated microRNA-30b-5p activates macrophages through the SIRT1/ NF-κB pathway in cell senescence

**DOI:** 10.3389/fimmu.2022.955175

**Published:** 2022-08-31

**Authors:** Yu Xiao, Jiaqi Liang, Kenneth W. Witwer, Ying Zhang, Qian Wang, Hang Yin

**Affiliations:** ^1^ Department of Laboratory Medicine, Zhujiang Hospital, Southern Medical University, Guangzhou, China; ^2^ School of Pharmaceutical Sciences, Tsinghua University, Beijing, China; ^3^ Tsinghua University-Peking University Joint Center for Life Sciences, Tsinghua University, Beijing, China; ^4^ Beijing Advanced Innovation Center for Structural Biology, Tsinghua University, Beijing, China; ^5^ Department of Molecular and Comparative Pathobiology, Baltimore, MD, United States; ^6^ Department of Neurology, The Johns Hopkins University School of Medicine, Baltimore, MD, United States

**Keywords:** extracellular vesicles, miRNA, SIRT1, cell senescence, NF-κB

## Abstract

Chronic inflammation is widely observed in aging, but it is unclear whether extracellular vesicles (EVs) play a role in chronic disease-associated senescence. In our study, LC/MS profiling revealed that senescent cell derived EVs (SEN EVs) activate the immune response pathways of macrophages. Significantly more EVs were found in the supernatant of SEN than of control (CON) cell cultures, and SEN EVs were enriched in miR-30b-5p, which directly target sirtuin1 (SIRT1). *In vitro*, we found that SEN EV treatment resulted in increased cellular levels of interleukin-1β (IL-1β) and IL-6 and decreased levels of SIRT1. Increased cytokine levels could be reversed by SIRT1 activation and miR-30b-5p inhibition. Furthermore, miR-30b-5p significantly increased with age in both mouse liver tissue and EVs harvested from the tissue, with differences in EVs observed both earlier and in the later magnitude of aging. Western blot and qPCR proved that miR-30b-5p downregulated the level of SIRT1 in mouse macrophages. Collectively, we propose that EVs carrying miR-30b-5p from SEN cells can induce chronic inflammation through macrophage activation. This occurs through the downregulation of SIRT1 and the corresponding activation of NF-κB pathways that enhance pro-inflammatory cytokine production. Collectively, these results demonstrate that EVs carrying pro-inflammatory signals are released by SEN cells and then activate immune cells in the SEN microenvironment, changing the inflammatory balance. Our results also explain why inflammation increases with age even though SEN cells can be immediately eliminated under rigorous immune surveillance.

## Introduction

The control and balance of cell senescence can regulate the occurrence and development of chronic diseases (1). During the process of aging, cells at different stages of cellular senescence (2) accumulate in tissues and secrete a large number of biologically active molecules, particularly pro-inflammatory cytokines, chemokines, and matrix remodeling enzymes that collectively contribute to the senescence-associated secretory phenotype (SASP) (3). Factors involved in SASP drive the systemic, low-grade, chronic inflammation that accompanies human aging (4). Short-term exposure to SASP stimulates the recruitment of immune cells to eliminate precancerous and senescent (SEN) cells, thereby preventing tumorigenesis; however, long-term exposure to SASP produces chronic inflammation and promotes tumorigenesis (5). In aging microenvironment, with the increasing investigation of the significant role of Extracellular vesicles (EVs), aging-associated EVs are now believed to play multiple complex roles in disease progression.

EVs can be released by a variety of cells and are believed to play a pivotal role in cell-cell communication both locally and remotely (6–13). These particles are thought to be effective circulating factors that regulate immune responses including inflammatory responses (14–16). Due to their lipid bilayers, EVs are readily phagocytosed by many different cell types (17, 18), especially immune cells, which makes it particularly important to study the influence of EVs and their contents on recipient cells. Recent studies have also revealed the functions of SEN cell-derived EVs (SEN EVs) (19). Specifically, EVs are widely reported in the aging, extracellular microenvironment and may transmit senescence signals in autocrine, paracrine, and endocrine ways like SASP (20).

Y RNA and tRNA fragments from EVs can trigger immune responses (16), and EVs microRNAs (miRNAs) have several reported roles in immune regulation. EV-miR-155 enhances the expression of pro-inflammatory cytokines, while EV-miR-146a attenuates inflammatory responses *via* dendritic cells, and macrophages uptake circulating EVs (17). Also, EV-miR-192, an immunomodulatory aging-associated microRNA, attenuated the hyperinflammatory state and improved vaccine efficacy in geriatric mice (21). Although major contributions to organismal aging have been demonstrated for EVs and their miRNA cargo, there is limited knowledge of the effect of cellular senescence on EVs contents and, in turn, on how EV-shuttled miRNAs might influence immune cells. However, circulating EVs concentrations seem to decline with age, possibly as a consequence of increased internalization by immune cells (22). Downregulated, inhibited, or defective activity of SIRT1 has been investigated in various cardiovascular (23, 24), renal (25), and aging-associated diseases (26, 27). SIRT1, as a type III histone/protein deacetylase, has many non-histone targets, such as p53 (28), FOXO (29), PGC1-α (30), NF-κB (31), which are involved in inflammation, cellular senescence, oxidative stress, energy metabolism, and DNA damage response (32). Among downstream targets of SIRT1, NF-κB is thought to be a major regulator of inflammation because it regulates the transcription of genes involved in establishing immune and inflammatory responses (24, 33, 34). Recent studies show that SIRT1 could be a regulatory element in the immune system, whose altered functions influence immune disorder disease development (35). Also, in a mouse lupus model, a SIRT1 activator effectively protected against disease progression (36). In the clinic, SIRT1 is well known to have anti-inflammatory properties (37–39).

In this study, we report that SEN EVs can induce transcription of pro-inflammatory cytokine genes in macrophages *via* downregulating SIRT1. We then evaluate several miRNAs that target the mRNA of SIRT1. Our results suggest that EV-associated miR-30b-5p reduces the levels of SIRT1 in recipient cells. In mouse aging and cell senescence processes, miR-30b-5p in EVs increases in a senescence degree-dependent fashion. Taken together, our study demonstrates that EV-carried miR-30b-5p regulates inflammatory responses of macrophages by downregulating SIRT1. A key challenge is to understand more precisely how EVs function in truly physiological settings. Our findings have revealed an EV-mediated delivery mechanism for miR-30b-5p, which reduces SIRT1 levels and counteracts NF-κB signaling, suggesting a potential avenue for anti-inflammatory intervention in humans.

## Materials and methods

### Cell lines and cell culture

An *in vitro* SEN EV functional assay was established using L929 cell lines that were cultured for 45 to 50 generations, and hyper SEN (h-SEN) cell lines were used etoposide-induced SEN cells. Non-senescent (control, CON) cells were used for comparison representing young cells. Raw 264.7 cells were used as the macrophage model. All cells were purchased from ATCC and cultured in Dulbecco’s Modified Eagle’s Medium (Gibco™, USA) supplemented with 10% (V/V) fetal bovine serum (FBS) (10099-141, Gibco™, USA) or EV-free FBS, 2 mM L-glutamine, 100 U/ml penicillin and 100 mg/ml streptomycin (Gibco™, USA). EV-free FBS was prepared by ultracentrifugation (Beckman Coulter, Optima XPN-100) at 120,000 × *g* for 12 h at 4 °C and filtration of the FBS supernatant with a 0.22 μm PVDF filter (Merck Millipore ltd). Cells were cultured at 37°C in a humidified incubator containing 5% CO_2_ and tested negative for mycoplasma infection every week.

Senescence was confirmed in the SEN and h-SEN models using the ratio of phospho-H2A.X to *β*-Actin, a DNA damage marker, (40, 41) and SASP levels.

Peripheral blood mononuclear cell (PBMC) were isolated from murine whole blood with Ficoll reagent following the previously established protocols (42).

Cell transfection was performed with Lipofectamine™ 3000 Transfection Reagent (L3000150, Invitrogen™, USA) according to the manufacturer’s protocols.

### Drug administration

The stock solution of SRT1720 (Sigma-Aldrich, St. Louis, MO, USA), a previously reported selective SIRT1 agonist (43), was prepared in dimethyl sulfoxide (DMSO) (D2438, Sigma-Aldrich) at storage concentration. The stock solutions were diluted by cell culture medium to the indicated concentrations prior to cell treatment.

### EVs isolation and characterization

The supernatant was collected from different groups of cells after 24-48 h culture and stored at 4 °C before EVs isolation within 72 h of harvest by differential ultracentrifugation (14, 44). Briefly, cell supernatants were firstly centrifuged at 2,000 × *g* for 15 min at 4 °C to remove floating dead cells and cell debris. Supernatants were gently transferred to a new tube and centrifuged at 12,000 × *g* for 45 min at 4 °C to pellet larger microvesicles and subcellular organelles. The medium was filtered through a 0.22 μm PVDF filter (Merck Millipore ltd) (44) before ultracentrifugation at 120,000 × *g* for 1 h. Then, the supernatant was discarded, followed by a washing step in PBS. Finally, the EVs were resuspended in 100 μL PBS or ddH2O.

To further characterize EVs, we used transmission electron microscopy (TEM), nanoparticle tracking analysis (NTA), and Western blots (WB) as recommended by the Minimal Information For Studies of Extracellular Vesicles (MISEV) guidelines developed by the International Society for Extracellular Vesicles in 2018 (45).

EVs (20 μL, ~10^7^ particles) suspended in ddH_2_O were loaded onto a copper grid and negatively stained with uranyl acetate solution for 30 seconds. The grid was then examined with an H7650B transmission electron microscope.

The particle size distribution and concentration of EVs samples were measured by a light-scattering-based NTA device (Malvern Instruments, United Kingdom) (46). To maximize the reliability of quantifications and size-distribution results, we diluted each EVs samples into the proper concentration range before measurement (47) and tracked the Brownian motion of laser-illuminated individual particles using cameral level 16 and detection threshold 7. Each sample was measured using three 60 s videos and analyzed by NanoSight NTA 3.1 software, which calculates particle diameter using the Stokes-Einstein equation and also analyzes concentration.

### Immunoblot analysis

For whole-cell and EVs proteins extraction, cells and EVs were firstly washed three times in ice-cold pH=7.4 PBS (10010094, Gibco™, USA) and solubilized in RIPA lysis buffer (P0013B, Beyotime, China) supplemented with protease inhibitor cocktails (78434, Thermo Scientific™, USA) on ice for 10 min. Samples were centrifuged at 12,000 × *g* at 4°C to remove cell debris. Sample lysates were quantified by Pierce BCA protein assay according to the manufacturer’s protocols (23235, Thermo Scientific™, USA). Protein samples were added with a proper volume of 6X protein loading buffer (DL101-02, TransGen Biotech, China) before protein denaturing. Proteins were separated by 8%-12% SDS–PAGE, electrotransferred to 0.45um PVDF membranes (Millipore, MA, USA) by SDS-PAGE gel transfer system (Bio-Rad, USA), and blocked with TBST containing 5% (w/v) skimmed milk powder (D8340, Solarbio, China) before incubation with primary antibodies at room temperature (RT) for 2 h or 4°C overnight and with 1:5000 diluted secondary antibodies for 1 h at RT. Protein immunoblots were detected by horseradish peroxidase-based ECL agent (34580, Thermo Scientific™, USA) for 5 min, and images were acquired with an iBright™ 1500 imaging system. Target protein levels were normalized by *β*-Actin level of the same samples.

Primary antibodies used in this study are listed below:

CD63 (1:1000) (rabbit monoclonal, ab59479, Abcam, Cambridge, UK), Alix (1:1000) (Proteintech, 12422-1-AP, USA), Calnexin (1:1000) (rabbit monoclonal, ab133615, Abcam, Cambridge, UK), SIRT1 (D1D7) (1:1000) (Rabbit mAb #9475, Cell Signaling Technology, Danvers, MA), Phospho-Histone H2A.X (Ser139) (20E3) (1:1000) (Rabbit mAb #9718, Cell Signaling Technology, Danvers, MA), *β*-Actin (13E5) (1:1000) (Rabbit mAb #4970, Cell Signaling Technology, Danvers, MA)

### Immunofluorescence staining

Cells were seeded on coverslips in 12-well plates and treated as indicated. After the supernatants were removed, the cells were washed with PBS for three times, then fixed with 4% paraformaldehyde (P1110, Solarbio, China) for 10 min, and permeabilized with 0.2% (v/v) Triton X-100 (T8200, Solarbio, China) after three times of washing, and blocked with 3% (w/v) BSA for 1 h. Then, the cells were incubated with 3% [w/v] BSA diluted anti-NF-κB p65 antibody (1:200) (recombinant antibody, 80979-1-RR, proteintech, USA) overnight at 4°C and incubated with 3% [w/v] BSA diluted Alexa Fluor 488-labeled goat anti-rabbit IgG (H+L) (1:2000) (A11034, Invitrogen™, USA) and DAPI for 1 h at RT after three washes. Finally, the coverslips were fixed on slides using a fluorescent mounting medium (HC08, Sigma-Aldrich, USA), and images were acquired with a Nikon A1RMP confocal microscope.

### Proteomics

Protein samples were separated by SDS-PAGE. In-gel digestion was carried out with sequencing-grade modified trypsin in 50 mM ammonium bicarbonate at 37°C overnight. The peptides were extracted twice with 0.1% trifluoroacetic acid in a 50% acetonitrile aqueous solution. Extracts were centrifuged in a speedvac to reduce the volume. Tryptic peptides were dissolved in 20 μl 0.1% TFA.

For LC-MS/MS analysis, the peptides were separated by Thermo-Dionex Ultimate 3000 HPLC system, which was directly interfaced with a Thermo Scientific Q Exactive mass spectrometer. The Q Exactive mass spectrometer was operated in the data-dependent acquisition mode using Xcalibur 2.1.2 software, and there was a single full-scan mass spectrum in the orbitrap (300-1800 m/z, 70,000 resolution) followed by 20 data-dependent MS/MS scans at 27% normalized collision energy (HCD).

Perseus software was used to analyze the data (48).

### Quantitative reverse-transcription PCR of mRNA and miRNAs

For qRT-PCR of mRNA, cells were seeded in 6-well plates and treated as indicated. Total RNA was collected with TRIzol reagent (15596018, Invitrogen ™, USA). The DEPC H_2_O diluted RNA was reverse transcribed using an iScript cDNA synthesis kit and analyzed by qPCR using iTaq Universal SYBR Green Supermix (in a Bio-Rad T100 thermal cycler). All reagents were used according to the manufacturer’s instructions. *β*-Actin was used as the internal control of mRNA and U6 was used as an internal normalization control of miRNAs.

The primers are listed below:

Mouse-*Il-1β*-F: GCAACTGTTCCTGAACTCAACT

Mouse-*Il-1β*-R: ATCTTTTGGGGTCCGTCAACT

Mouse-*Il-6*-F: TAGTCCTTCCTACCCCAATTTCC

Mouse-*Il-6*-R: TTGGTCCTTAGCCACTCCTTC

Mouse-*Sirt1*-F: ATGACGCTGTGGCAGATTGTT

Mouse-*Sirt1*-R: CCGCAAGGCGAGCATAGAT

Mouse-*β-Actin*-F: TGACGTTGACATCCGTAAAGACC

Mouse-*β-Actin*-R: AAGGGTGTAAAACGCAGCTCA

Mouse- *Il-8*-F: CAAGGCTGGTCCATGCTCC

Mouse- *Il-8*-R: TGCTATCACTTCCTTTCTGTTGC

Mouse-p16-F: CGCAGGTTCTTGGTCACTGT

Mouse-p16-R: TGTTCACGAAAGCCAGAGCG

Mouse-p21-F: CCTGGTGATGTCCGACCTG

Mouse-p21-R: CCATGAGCGCATCGCAATC

Mouse-TP53-F: CCATGAGCGCATCGCAATC

Mouse-TP53-R: CGGAACATCTCGAAGCGTTTA

For qRT-PCR of miRNA, total RNA of cells or EVs was extracted with TRIzol reagent (15596018, Invitrogen ™, USA). The DEPC H_2_O diluted RNA was reverse transcribed using an iScript cDNA synthesis kit supplemented with stem-loop RT primers. qPCR analysis was conducted using iTaq Universal SYBR Green Supermix in a Bio-Rad T100 thermal cycler. ADD normalization strategy.

The primers are listed below:

mmu-miR-30b-5p-RT(stem): GTCGTATCCAGTGCAGGGTCCGAGGTATTCGCACTGGATACGACAGCTGA

mmu-miR-30b-5p-F(stem): GCGCTGTAAACATCCTACAC

U6-F: CTCGCTTCGGCAGCACA

U6-R: AACGCTTCACGAATTTGCGT

Universal-R: GCGATCACATTGCCAGGG

The sequences used for miRNA mimics and antagonists are listed below:

mmu-miR-30b-5p mimics:

sense: 5’-UGUAAACAUCCUACACUCAGCU-3’

anti-sense 5’-CUGAGUGUAGGAUGUUUACAUU-3’

mmu-miR-30b-5p antagonist: 5’-AGCUGAGUGUAGGAUGUUUACA-3’

### Dual-luciferase reporter assay

pGLO vector-SIRT1-wild type and pGLO-SIRT1-mutant reporter plasmid vectors were constructed by integrating target fragments of miR-30b-5p. SIRT1-WT or SIRT1-MUT was co-transfected with miR-30b-5p mimics for 48 h. And then measured the luciferase activity with the Dual-Lumi™ Luciferase Reporter Assay Kit (RG088S, Beyotime, China).

### Statistical analyses

The data are presented as group mean ± SD. Unpaired Student’s t-tests were used to analyze two-group comparisons, and one-way ANOVA for more than two groups, followed by Bonferroni’s *post-hoc* test, using GraphPad Prism 6.0. Group differences at the level of *p* < 0.05 were considered to be statistically significant. “*” represents *p* < 0.05; “**” represents *p* < 0.01, and “***” represents *p* < 0.001. "ns" denotes not significant versus control.

## Results

### SEN EVs induce macrophage immune responses

**Figure 1:**
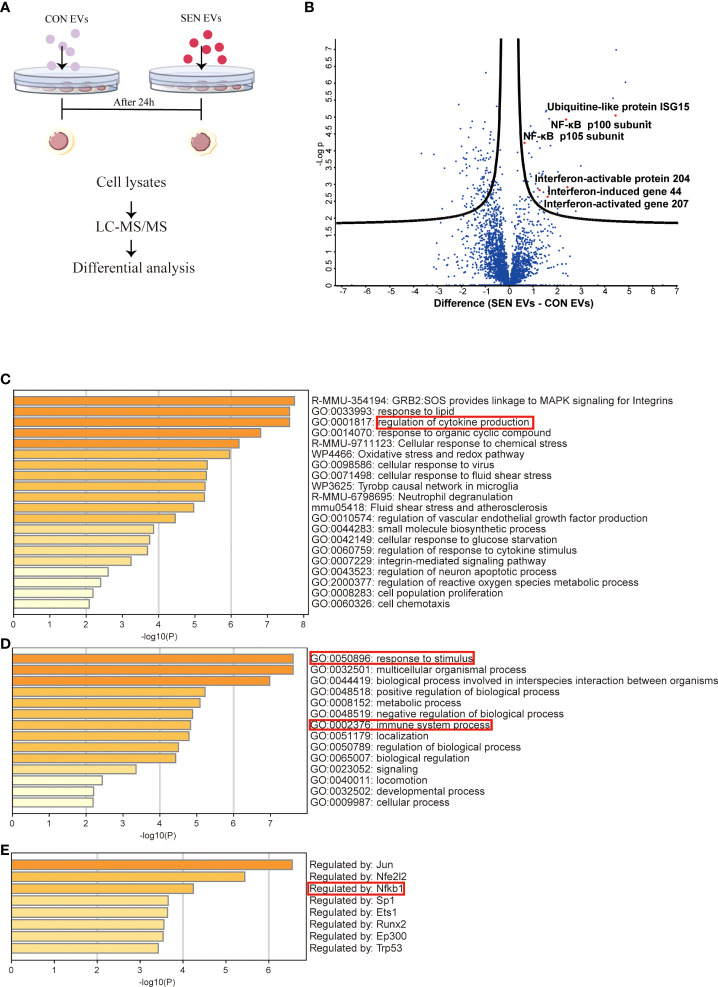
Proteomics study of Raw 264.7 cells treated with CON and SEN EVs. **(A)** Workflow used for proteomic analysis. **(B)** Volcano plot of differentially expressed proteins; dots above the black curves represent proteins with differences with False Discovery Rate (FDR) < 0.025. Bar graphs of enriched terms **(C)** across input gene lists; **(D)** with GO terms only; and **(E)** by TRRUST. Data were from four independent repeats.

Since EVs mediate crucial cell-cell communication (49, 50), we aimed to assess the function of EVs at an early stage of senescence. Raw 264.7 cells were co-cultured for 24 h with EVs from SEN and CON cells (**Figure 1A**). Cellular protein content extracted from co-cultured cells was then assessed with liquid chromatography-tandem mass spectrometry (LC-MS/MS). In total, 2939 unique proteins were identified (**Figure S1C**). These are shown by hierarchical clustering (**Figure S1A**). In addition, a volcano plot was prepared to visualize differentially expressed proteins (**Figure 1B**). Based on false discovery rate cutoffs of 0.025, 157 protein were identified as differentially expressed between Raw 264.7 cells treated with CON EVs and SEN EVs, including 48 up-regulated proteins (see heatmap, Figure S1D). We were interested to observe that SIRT1 decreased in SEN EV-treated cells (**Figure S1D**). By protein categorization by Metascape (51), we first identified all statistically enriched terms, including Gene Ontology (GO), Kyoto Encyclopedia of Genes and Genomes (KEGG), canonical pathways, and hallmark gene sets. Accumulative hypergeometric *p*-values and enrichment factors were calculated and used for filtering (**Figure 1C**). Next, we did GO analysis for the biological process subclass and found enriched proteins related to the immune system (**Figure 1D**). Finally, Transcriptional Regulatory Relationships Unraveled by Sentence-based Text mining (TRRUST) (**Figure 1E**) revealed that, among others, inflammation-related proteins and the NF-κB signaling pathway were enriched in the cellular proteome after SEN EVs treatment. The network of these enriched proteins is shown in **Figures S1A, B**. Taken together, our proteomics results and knowledge of pathways involved in senescence prompted us to further investigate the possible role of SEN EVs in influencing the NF-κB mediated inflammation pathway.

### SEN EVs regulate SIRT1 and induce canonical NF-κB activation

**Figure 2:**
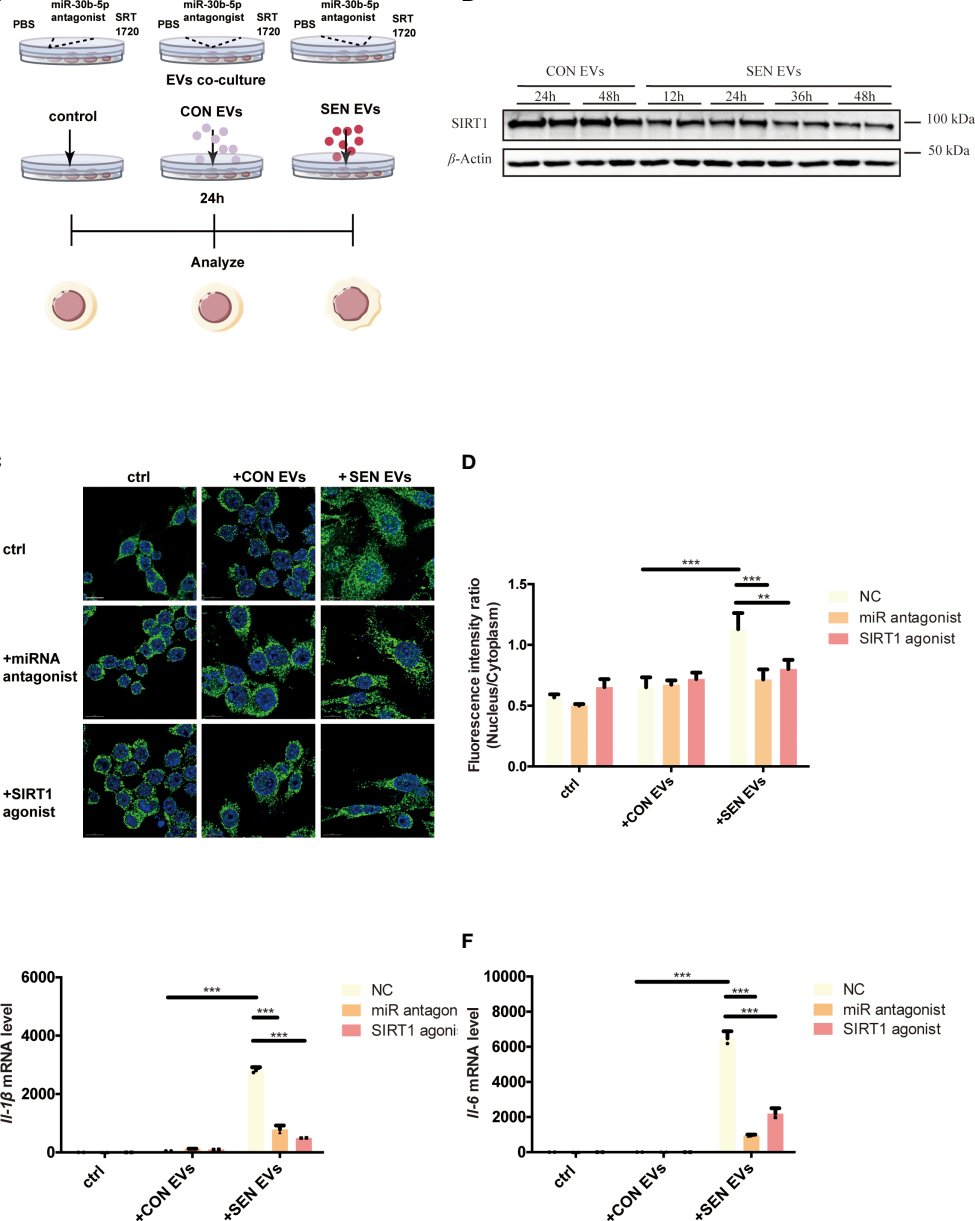
SIRT1 regulates NF-κB activation induced by SEN EVs. **(A)** Workflow of the experimental design. Cells were treated with PBS (Control, ctrl), CON, or SEN EVs after being pretreated with PBS, miR-30b-5p antagonist, or SRT1720. **(B)** Immunoblot analysis of SIRT1 protein level of cells treated with CON or SEN EVs for a time course. **(C)** Immunofluorescence analysis of DAPI (blue) and p65 (green) in Raw 264.7 cells. Scale bars: 5 μm. **(D)** Quantification of immunofluorescence ratio of p65 (Nucleus/cytoplasm). RT-qPCR analysis of **(E)** *Il-1β* and **(F)** *Il-6* mRNA levels of Raw 264.7 cells treated with PBS, CON, or SEN EVs and the indicated inhibitors. Data are from at least three independent experiments and are presented as the mean ± SD. **p<0.01; and ***p<0.001.

SIRT1 is a histone and non-histone deacetylase that is widely present in the nucleus and cytoplasm and can regulate the NF-κB signaling pathway (31). To test the hypothesis that SEN EVs may regulate SIRT1/NF-κB signaling, we constructed an EV co-culture system (**Figure 2A**). Having identified the NF-κB signaling pathway in our proteomics study, we found that the level of SIRT1 decreased after being co-cultured with SEN EVs in a time-dependent manner (**Figure 2B**). We hypothesized that it might be the upstream protein SIRT1, which can deacetylate NF-κB and thus inhibits activation of the NF-κB signaling pathway (52) and related inflammation. We further hypothesized that the decrease in SIRT1 is due to EV-associated miRNAs that target SIRT1 in Raw 264.7 macrophage cells allowing NF-κB-related inflammatory responses to tip the balance between pro-inflammatory and anti-inflammatory cytokines. We attempted to test ourthe hypotheses in the cell system shown in **Figure 2A**. Through fluorescence microscopy, the RelA/p65 subunit of NF-κB which is involved in canonical signaling was found to enter the nucleus of cells exposed to SEN EVs. In contrast, treating cells with a SIRT1 agonist decreased the amount of p65 nuclear entry (**Figures 2C, D**). We also tested the pro-inflammatory functions of SEN EVs by measuring cytokines. mRNA levels of pro-inflammatory cytokines IL-1β and IL-6 were increased by the addition of SEN EVs, but this increase was abrogated by treatment with a miRNA antagonist of miR-30b-5p and a SIRT1 agonist (**Figures 2E, F**).

### miR-30b-5p can target SIRT1 in cells

**Figure 3:**
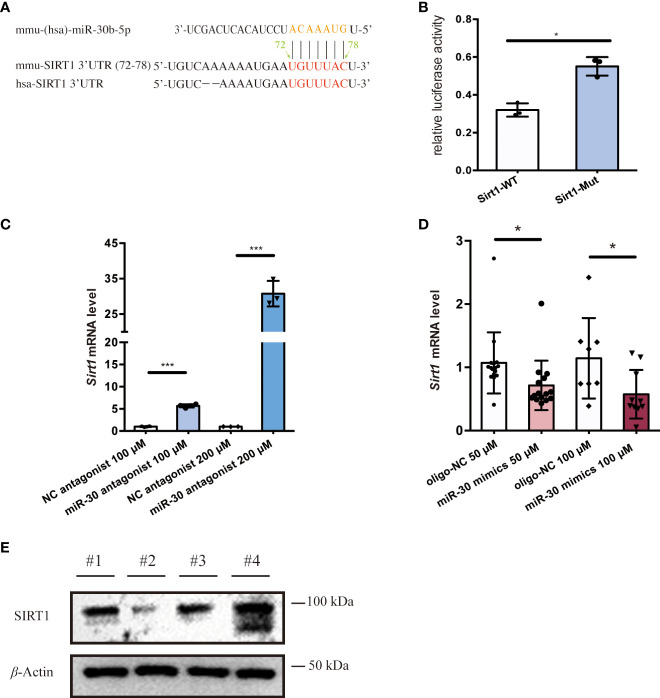
miR-30b-5p directly targets SIRT1 in macrophages. **(A)** miR-30b-5p target sequence in the 3’UTR of Sirt1. **(B)** Relative activity of firefly luciferase and *Renilla* luciferase after co-transfection with mimics and plasmids expressing wild-type or mutated target sites. **(C)** RT-qPCR analysis of *Sirt1* mRNA level of cells treated with the miR-30b-5p antagonist. **(D)** RT-qPCR analysis of *Sirt1* mRNA level of cells treated with miR-30b-5p mimics. **(E)** Immunoblot analysis of SIRT1 protein level of cells treated with miR-30b-5p mimics and antagonists. #1:mimics control group; #2: miR-30b-5p mimics group; #3: antagonists control group; #4: miR-30b-5p antagonists group. The data represent at least three independent experiments and are presented as mean ± SD. *p<0.05; and ***p<0.001.

To investigate EV cargo that might regulate the activation of macrophages, we utilized the TargetScan and ENCORI prediction tools to identify possible miRNA regulators of *Sirt1* mRNA. Among the candidate miRNAs, miR-30b-5p target the 3’UTR region of the *Sirt1* mRNA in both humans and mice (**Figure 3A**) and have relatively high prediction scores (**Figure S2A**). Also, miR-30 (53, 54) have been widely reported to be detected in EVs and are closely related to the progression of diverse diseases (54–56). To further investigate the role of miR-30b-5p in NF-κB mediated inflammation, we sought to verify *Sirt1* as a predicted target mRNA of this miRNA. Therefore, we set out to verify this target *in vitro*. We transfected Raw 264.7 cells with the miR-30b-5p anti-sense antagonist and found a significant upregulation of the mRNA level of *Sirt1* in cells (**Figure 3C**). Meanwhile, transfecting Raw 264.7 cells with miR-30b-5p mimics downregulated the mRNA level of *Sirt1* in cells (**Figure 3D**). These effects were also dose-dependent. In addition, the immunoblot assay showed that transfection of the antagonist and mimics of miR-30b-5p regulated the protein level of *SIRT1* in Raw 264.7 cells (**Figure 3E**). In conclusion, the mRNA of *Sirt1* may be an important target of miR-30b-5p in cells. Co-culturing macrophages with the antisense sequence of miR-30b-5p as the antagonist (**Figures 2C-F**) can reverse the pro-inflammatory function of SEN EVs, and the transfection of 10 nM miR-30b-5p caused a remarkable inhibition of cell inflammatory compared with that of the control group (**Figures 2E, F**). Together, these results indicate that the functions of SEN EVs may include SIRT1-downregulating effects of EV miR-30b-5p.

### miR-30b-5p level in cells and EVs are correlated with aging

**Figure 4:**
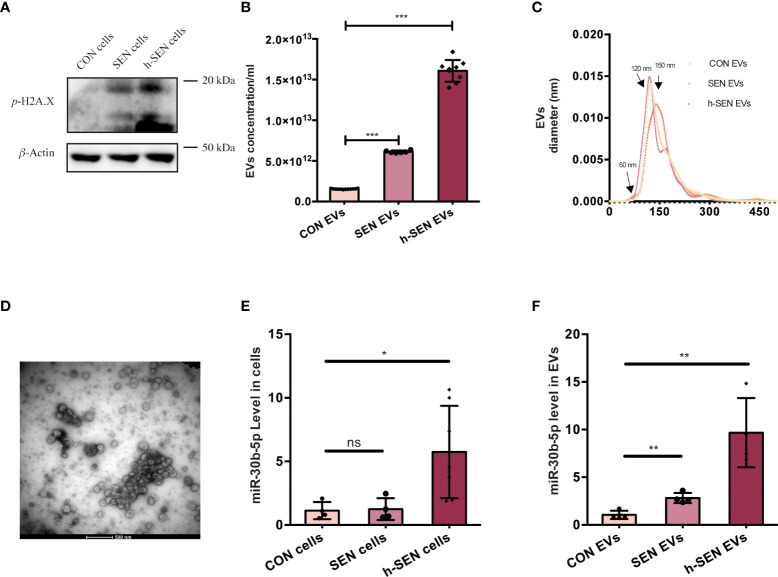
Characterization of CON and SEN cells and derived EVs. **(A)** Immunoblot of *p*-H2A.X/*β*-actin of CON, SEN, and h-SEN cell models. 10 μg proteins were loaded in each gel lane. **(B)** Particle concentrations and **(C)** particle size distributions of EVs preparations were determined by nanoparticle tracking analysis (NTA). **(D)** Transmission electron microscopy (TEM) micrograph of representative isolated SEN EVs. RT-qPCR result of miR-30b-5p levels of CON, SEN, and h-SEN cells **(E)** and EVs **(F)**. The data are from at least three independent experiments and are presented as mean ± SD. *p<0.05; **p<0.01; and ***p<0.001; ns denotes not significant versus control..

Hypothesizing that these miRNAs play a role in SEN EV-induced inflammation, we accordingly measured miRNA levels in cells and their EVs at several stages of senescence. Cell senescence was quantitated in CON, SEN, and h-SEN cells using the phospho-HA.X/*β*-Actin ratio (40, 41) (**Figure 4A**). The morphology and SASP level of CON, SEN, and h-SEN cells is shown in **Figures S3A-B**. NTA and TEM results for EVs secreted from CON, SEN, and h-SEN cells indicated that particle count in EVs preparations was positively correlated with senescence degree (**Figures 4B, C**). Also, h-SEN EVs have more EVs in a small, 60-nm subcluster, which might be more easily internalized by cells. Although EVs are rich in miRNAs and RNA binding proteins such as hnRNPA2B1 have been reported to sort miRNAs into EVs (57, 58), it is as yet unclear if or how miR-30b-5p is sorted. However, RT-qPCR of miR-30b-5p shows increased levels in cells and EVs, and that miRNA levels are positively correlated with senescence degree (**Figures 4E, F**).

### Morphology of adult and aging mouse liver EVs

**Figure 5:**
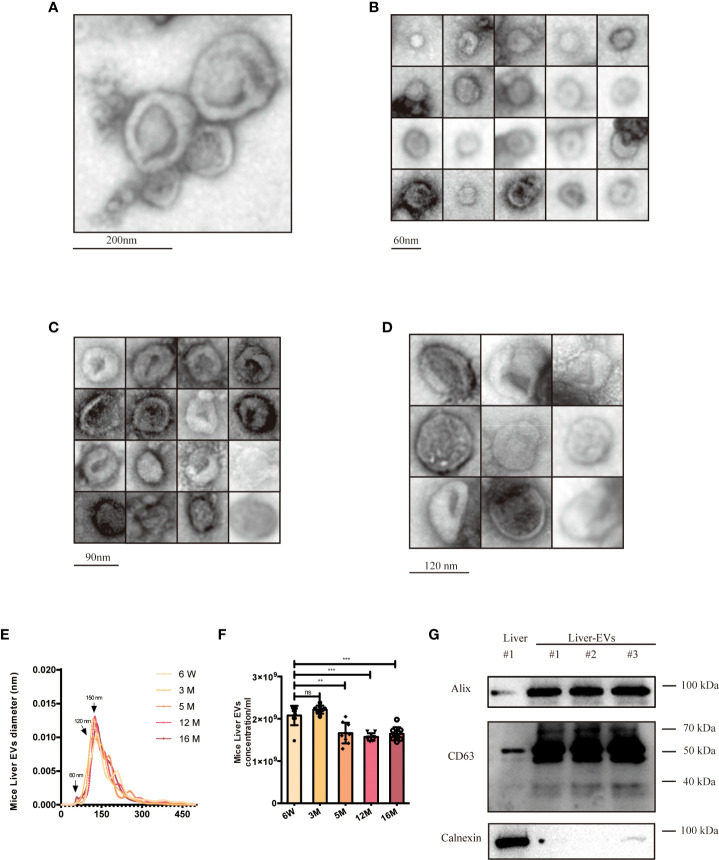
Characterization of young (6-week old) and aged (16-month old) mice liver EVs. **(A)** TEM analysis of EV clusters; scale bars: 200 nm. **(B)** TEM analysis of small EVs (60 nm). scale bars: 60 nm. **(C)** TEM analysis of EVs with diameter of ~80-90 nm; scale bars: 90 nm. **(D)** TEM analysis of EVs at a diameter of ~120 nm. scale bars; 120 nm. NTA size distributions **(E)** and particle concentrations **(F)** of liver EV preparations from mice at six ages as indicated. **(G)** Western blot of Alix, CD63, and Calnexin from liver EVs and liver tissues. The data represent at least three independent experiments and are presented as mean ± SD. **p<0.01; and ***p<0.001; ns denotes not significant versus control..

Livers were harvested from mice of different ages and used to prepare tissue and isolate tissue EVs. The latter were characterized by following established guidelines (45) (**Figure 5**). We used electron microscopy to reveal the morphology of isolated EVs, specifically definition by a lipid bilayer and a size of 30–150 nm (**Figure 5A**). Size-based EVs subclusters were measured and displayed (**Figures 5B-D**), and size profiles and concentrations of EVs from the liver were measured by NTA. By NTA, particle mode diameters ranged mostly from 120-160 nm, both in adult and different aging liver EVs samples (**Figure 5E**). It has been reported that SEN cells generally secrete more EVs than CON cells (59). Interestingly, we observed that particle counts in the liver tissue did not increase. By contrast, the EV levels decreased while the mice aged (**Figure 5F**), which contradicted with the previous reports, opening up new possibilities for the study of tissue-derived EVs. As reported, EVs of different sizes have different architectural features and uptake rates, and EVs of small sizes are more easily taken up by target cells (60). A larger subcluster of 60-nm EVs was observed in the 18-week (18W) group, while almost no 60-nm EVs were observed in the 6-week (6W) group. There is a speculation that these smaller EVs (60nm) produced during aging might contain more pro-inflammatory miRNAs, but this hypothesis cannot be confirmed at present due to technical limitations. Finally, several EVs marker proteins (CD63 and Alix) were detected from isolated EVs from different groups of mouse livers (**Figure 5G**) by immunoblotting, while calnexin, a cellular marker, was undetected or greatly depleted in EVs preparations.

### Levels of EV miR-30b-5p increased significantly with age in mice

**Figure 6:**
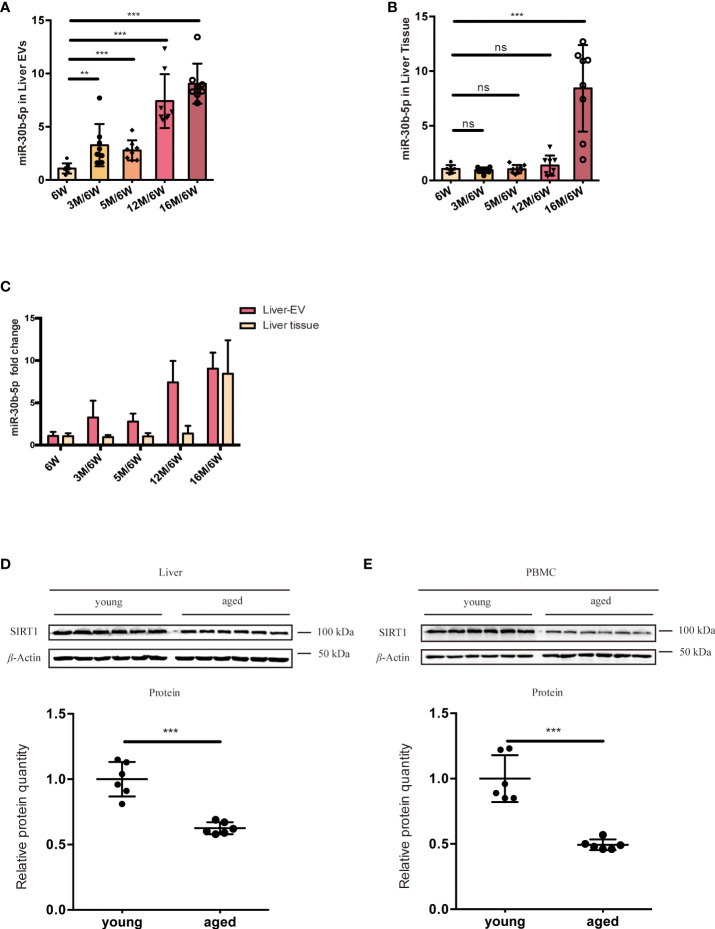
miR-30b-5p increase with aging in liver tissue and liver tissue EVs. **(A)** RT-qPCR of miR-30b-5p levels of mouse liver EVs. **(B)** RT-qPCR of miR-30b-5p levels from mouse liver tissue. **(C)** Comparison of miR-30b-5p levels from liver EVs and tissue. **(D)** Immunoblotting analysis of SIRT1 protein level of young (6-week old) and aged (16-month old) group ouse liver. 6 mice were randomly selected from each group. **(E)** Immunoblot analysis of SIRT1 protein level of young (6-week old) and aged (16-month old) group mouse PBMCs. 6 mice were randomly selected from each group. The data represent at least three independent experiments and are presented as mean ± SD. **p<0.01; and ***p<0.001, ns denotes not significant versus control. 6W: 6-week-old mice, 3M, 5M, 12M, and 16M: 3, 5, 12, and 16-month-old mice.

To find the source tissues of elevated miR-30b-5p, we measured heart, lung, spleen, along with liver tissue-derived EVs. We found that this miRNA increased with age in the liver. Next, we turned to the RNA extracted from different groups of mouse liver EVs and liver tissue and investigated the relationship between senescence and levels of EVs miRNA in the mouse model. The level of miR-30b-5p in mouse liver and liver tissue-derived EVs were measured (**Figures 6A-C**). The results showed that EV-derived miR-30b-5p increased with aging (**Figure 6A**). EV-associated miRNAs were increased already at 3 months, while in liver tissue, increases in miR-30b-5p were not discernible until 16 months, respectively (**Figure 6B**). We directly compared the miRNA levels between indicated EVs and cells, and the magnitude of the difference was also greater in EV preparations than in liver tissue (**Figure 6C**). To better observe the relationship between miR-30b-5p and SIRT1 in mouse liver, we assessed SIRT1 levels of liver tissues and PBMC in young and aging mouse populations (**Figures 6D, E**).

## Discussion

**Figure 7:**
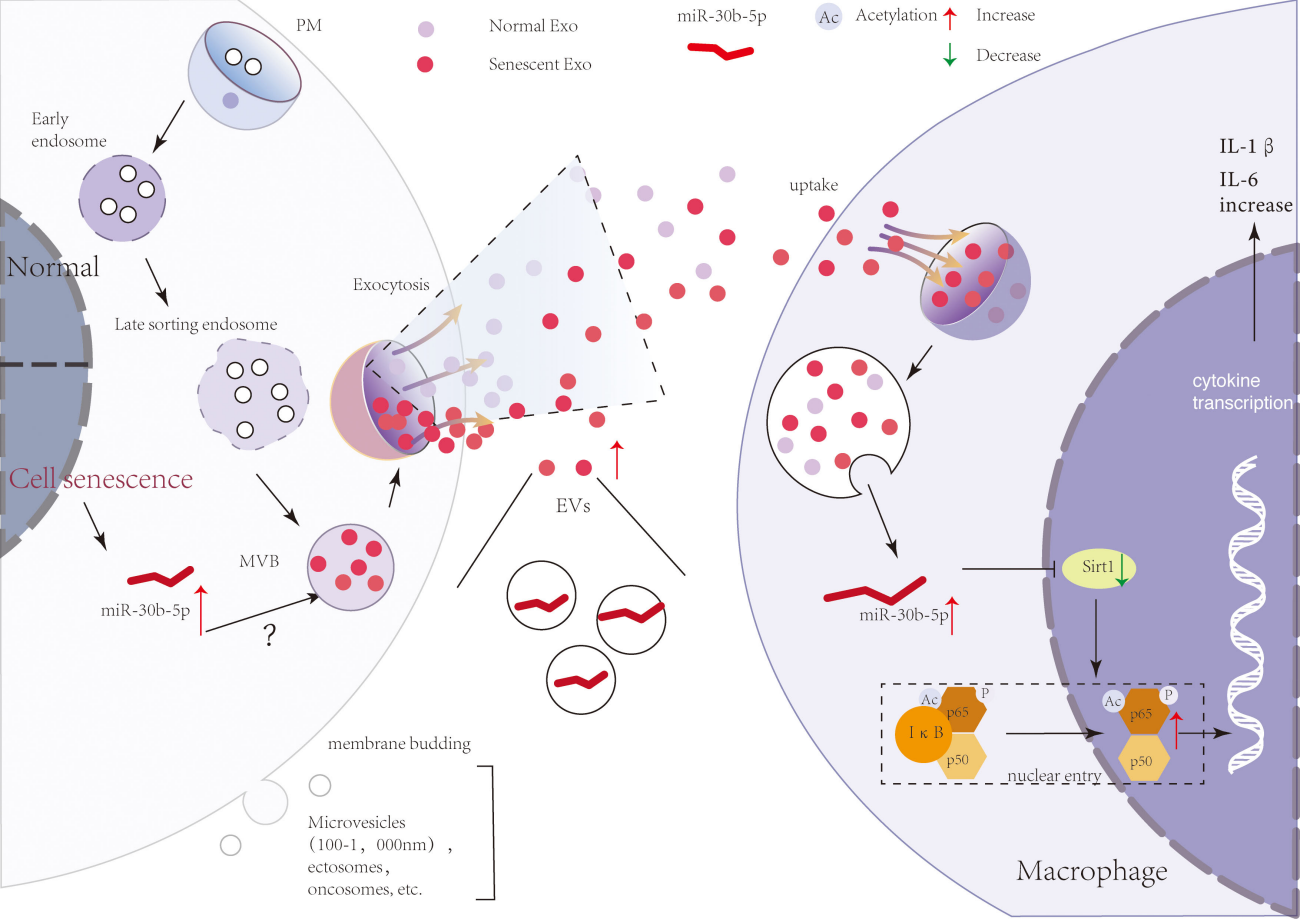
A brief model describing a theoretical mechanism whereby SEN cell-derived EVs transport pro-inflammatory signals to macrophages. SEN cells release more EVs that contain miR-30b-5p. With the uptake of SEN EVs by macrophages, miR-30b-5p is released from EVs and reduces the level of SIRT1, thereby mediating the increase of p65 entry into the nucleus, and then increasing the synthesis of cytokines IL-6 and IL-1β.

In natural senescence, previously reported evidence supports a major contribution of secreted EVs to the effects of SEN cells on their micro-environment (20, 61). In our study, we confirmed and extended these findings, showing that SEN EVs transport pro-inflammatory signals, but not pro-senescence signals directly, to recipient macrophages. A model of the mechanism is shown in **Figure 7**. SEN cells indeed released a significantly higher level of small (60-nm) EVs compared with normal cells. Importantly, these EVs were released relatively early in the process. Our data agree with the results of previous studies, including a mouse model of oncogene-induced senescence and human lung fibrotic lesions enriched in SEN cells (50) and bone marrow stromal cells (62).

Our findings support the hypothesis that SEN EVs are enriched in a group of “pre-aging” microRNAs that are transferred to recipient macrophages and influence important biological pathways. , although it remains to be shown what level of transported miRNA is necessary to induce changes in recipient cells. Analyzing the candidate miRNAs of EVs that could be involved in these effects, we found that miR-30b-5p directly targets the mRNA of Sirt1 and is more abundant in SEN EVs in vivo and in vitro. This miRNA is closely related to tumor and inflammation. Our results reveal that the early function of SEN EVs is mainly pro-inflammatory via the SIRT1/NF-κB signaling pathway, different from other reports that emphasize the pro-senescence function of SEN EVs. Indeed, also supporting a role for EV miRNAs, SEN EVs are reportedly enriched in miR-21-5p and miR-217, which were over-expressed in SEN cells and were capable of targeting not just SIRT1, but also DNMT1, another key enzyme in methylation pattern maintenance (63, 64).

To further elucidate the function of SEN EVs and understand if we could potentially manipulate them to change biological processes, we employed multiple regulators to treat recipient cells. Both SIRT1 agonist and miR-30b-5p anti-sense antagonist ameliorated processes associated with the pro-inflammatory function of SEN EVs. Encouragingly, these findings suggest that interventions against miRNAs in SEN cells and/or SEN EVs may be feasible.

In aged humans, levels of extracellular miR-30b-5p have been reported to be related with aging processes, albeit with apparently opposite results in different studies (41–43). In our study, we first addressed the EVs miR-30b-5p level and raise several questions that may be addressed in future studies. For example, we observed differences in the size distribution of EVs released from SEN cells, but whether miR-30b-5p are enriched in specific size classes of EVs is still unknown. It would also be interesting to investigate whether different size classes of EVs are different not just in content, but also in cell uptake or fusion abilities in this model, much as previously reported elsewhere (50). The topology of miRNAs, in or on EVs, might also be analyzed. Finally, we cannot fully rule out additional contributions of non-EV or non-miRNA effectors to the transferred senescence phenomenon.

Altogether, we provide evidence that during progression from a pre-senescence to a hyper-senescence stage, SEN cells can spread their miRNA signature, thus contributing to the development of a pro-inflammatory environment for immune cells. These microRNAs can be selectively sorted into and delivered through EVs. Also, our results indicate that EV-miR-30b-5p might be used as a pre-aging or early aging biomarkers to track aging trajectories. In conclusion, our finding may lay the foundation for further research, in particular to understanding how pro-inflammatory and pro-senescence signals carried by EVs can become druggable targets that can help modulate the immune-regulating and aging process and delay aging-associated disease development.

In conclusion, cellular senescence is a state of permanent cell-cycle arrest, the causes of which are incompletely understood. Learning more about the cellular and molecular contributors to senescence will facilitate new approaches to treating senescence-related phenomena and diseases. Our work here reveals an unappreciated relationship between innate immune responses and senescence, establishing a link between cells and different cell types that involves cellular EVs transfer of miRNAs. These results give new insights into the inflammatory process in SASP and therefore heighten our understanding of the accurate regulation of senescence. SEN EV-induced inflammatory responses of macrophages may also be persistent: the macrophages will not be cleared by immune cells, since these SEN cells, possibly influenced chronically by released SEN EVs, are in a state of immune escape. The sustained action of SEN EVs may thus assist in transforming the macrophage into a promoter of aging and disease.

## Data availability statement

The datasets presented in this study can be found in online repositories. The names of the repository/repositories and accession number(s) can be found in the article/**Supplementary Material**.

## Ethics statement

The animal study was reviewed and approved by Laboratory Animal Resources Center at Tsinghua University.

## Author contributions

The corresponding author HY and QW contributed to the conception and design of the study. YX designed all experiments. YX, JL, and YZ performed all experiments. HY, KW, and QW have made great contributions to the manuscript guidance. All authors contributed to the article and approved the submitted version.

## Funding

This work was supported by funds from the National Natural Science Foundation of China (Grant No. 21825702, 22137004, 82072334, and 21977061), Beijing Advanced Innovation Center for Structural Biology Funding (Grant No. 20151551402), Beijing Outstanding Young Scientist Program (Grant No. BJJWZYJH01201910003013).

## Acknowledgments

We thank all the laboratory secretaries for their support in the operation of the laboratory. We thank the Protein Research Technology Center at Tsinghua University for the LC-MS/MS instruments utilization and data analysis guidance. We thank the State Key Laboratory of Membrane Biology at Tsinghua University for permission to utilize ultracentrifuges and other instruments. We thank the Laboratory Animal Resources Center at Tsinghua University for the experimental animal breed. We thank the Central Laboratory at Southern Medical University for instrument use training and instruction. We thank the funding from the National Natural Science Foundation of China and other affiliations.

## Conflict of interest

The authors declare that the research was conducted in the absence of any commercial or financial relationships that could be construed as a potential conflict of interest.

## Publisher’s note

All claims expressed in this article are solely those of the authors and do not necessarily represent those of their affiliated organizations, or those of the publisher, the editors and the reviewers. Any product that may be evaluated in this article, or claim that may be made by its manufacturer, is not guaranteed or endorsed by the publisher.

## References

[1] Bussian TJ Aziz A Meyer CF Swenson BL van Deursen JM Baker DJ. Clearance of senescent glial cells prevents tau-dependent pathology and cognitive decline. Nature (2018) 562(7728):578–82. doi: 10.1038/s41586-018-0543-y PMC6206507 30232451

[2] Ogrodnik M. Cellular aging beyond cellular senescence: Markers of senescence prior to cell cycle arrest *in vitro* and *in vivo* . Aging Cell (2021) 20(4):e13338. doi: 10.1111/acel.13338 33711211 PMC8045927

[3] Coppe JP Patil CK Rodier F Sun Y Munoz DP Goldstein J. Senescence-associated secretory phenotypes reveal cell-nonautonomous functions of oncogenic RAS and the p53 tumor suppressor. PloS Biol (2008) 6(12):2853–68. doi: 10.1371/journal.pbio.0060301 PMC2592359 19053174

[4] Franceschi C Bonafe M Valensin S Olivieri F De Luca M Ottaviani E. Inflamm-aging. an evolutionary perspective on immunosenescence. Ann N Y Acad Sci (2000) 908:244–54. doi: 10.1111/j.1749-6632.2000.tb06651.x 10911963

[5] Loo TM Miyata K Tanaka Y Takahashi A. Cellular senescence and senescence-associated secretory phenotype *via* the cGAS-STING signaling pathway in cancer. Cancer Sci (2020) 111(2):304–11. doi: 10.1111/cas.14266 PMC7004529 31799772

[6] Thery C Zitvogel L Amigorena S. Exosomes: composition, biogenesis and function. Nat Rev Immunol (2002) 2(8):569–79. doi: 10.1038/nri855 12154376

[7] Thery C Ostrowski M Segura E. Membrane vesicles as conveyors of immune responses. Nat Rev Immunol (2009) 9(8):581–93. doi: 10.1038/nri2567 19498381

[8] Gurunathan S Kang MH Jeyaraj M Qasim M Kim JH. Review of the isolation, characterization, biological function, and multifarious therapeutic approaches of exosomes. Cells (2019) 8(4). doi: 10.3390/cells8040307 PMC6523673 30987213

[9] Raposo G Stoorvogel W. Extracellular vesicles: exosomes, microvesicles, and friends. J Cell Biol (2013) 200(4):373–83. doi: 10.1083/jcb.201211138 PMC3575529 23420871

[10] Holme PA Solum NO Brosstad F Roger M Abdelnoor M. Demonstration of platelet-derived microvesicles in blood from patients with activated coagulation and fibrinolysis using a filtration technique and western blotting. Thromb Haemost (1994) 72(5):666–71. 7900071

[11] Hess C Sadallah S Hefti A Landmann R Schifferli JA. Ectosomes released by human neutrophils are specialized functional units. J Immunol (1999) 163(8):4564–73. 10510400

[12] Cocucci E Racchetti G Meldolesi J. Shedding microvesicles: artefacts no more. Trends Cell Biol (2009) 19(2):43–51. doi: 10.1016/j.tcb.2008.11.003 19144520

[13] Gyorgy B Modos K Pallinger E Paloczi K Pasztoi M Misjak P. Detection and isolation of cell-derived microparticles are compromised by protein complexes resulting from shared biophysical parameters. Blood (2011) 117(4):e39–48. doi: 10.1182/blood-2010-09-307595 21041717

[14] Colombo M Raposo G Thery C. Biogenesis, secretion, and intercellular interactions of exosomes and other extracellular vesicles. Annu Rev Cell Dev Biol (2014) 30:255–89. doi: 10.1146/annurev-cellbio-101512-122326 25288114

[15] Robbins PD Morelli AE. Regulation of immune responses by extracellular vesicles. Nat Rev Immunol (2014) 14(3):195–208. doi: 10.1038/nri3622 24566916 PMC4350779

[16] Xiao Y Driedonks T Witwer KW Wang Q Yin H. How does an RNA selfie work? EV-associated RNA in innate immunity as self or danger. J Extracell Vesicles (2020) 9(1):1793515. doi: 10.1080/20013078.2020.1793515 32944182 PMC7480420

[17] Alexander M Hu R Runtsch MC Kagele DA Mosbruger TL Tolmachova T. Exosome-delivered microRNAs modulate the inflammatory response to endotoxin. Nat Commun (2015) 6:7321. doi: 10.1038/ncomms8321 26084661 PMC4557301

[18] Valadi H Ekstrom K Bossios A Sjostrand M Lee JJ Lotvall JO. Exosome-mediated transfer of mRNAs and microRNAs is a novel mechanism of genetic exchange between cells. Nat Cell Biol (2007) 9(6):654–9. doi: 10.1038/ncb1596 17486113

[19] Horowitz AM Fan X Bieri G Smith LK Sanchez-Diaz CI Schroer AB. Blood factors transfer beneficial effects of exercise on neurogenesis and cognition to the aged brain. Science (2020) 369(6500):167–73. doi: 10.1126/science.aaw2622 PMC7879650 32646997

[20] Yin Y Chen H Wang Y Zhang L Wang X. Roles of extracellular vesicles in the aging microenvironment and age-related diseases. J Extracell Vesicles (2021) 10(12):e12154. doi: 10.1002/jev2.12154 34609061 PMC8491204

[21] Tsukamoto H Kouwaki T Oshiumi H. Aging-associated extracellular vesicles contain immune regulatory microRNAs alleviating hyperinflammatory state and immune dysfunction in the elderly. iScience (2020) 23(9):101520. doi: 10.1016/j.isci.2020.101520 32927264 PMC7495115

[22] Eitan E Green J Bodogai M Mode NA Baek R Jorgensen MM. Age-related changes in plasma extracellular vesicle characteristics and internalization by leukocytes. Sci Rep (2017) 7(1):1342. doi: 10.1038/s41598-017-01386-z 28465537 PMC5430958

[23] Karbowska M Kaminski TW Znorko B Domaniewski T Misztal T Rusak T. Indoxyl sulfate promotes arterial thrombosis in rat model *via* increased levels of complex TF/VII, PAI-1, platelet activation as well as decreased contents of SIRT1 and SIRT3. Front Physiol (2018) 9:1623. doi: 10.3389/fphys.2018.01623 30546314 PMC6279869

[24] Breitenstein A Stein S Holy EW Camici GG Lohmann C Akhmedov A. Sirt1 inhibition promotes *in vivo* arterial thrombosis and tissue factor expression in stimulated cells. Cardiovasc Res (2011) 89(2):464–72. doi: 10.1093/cvr/cvq339 20978007

[25] Vasko R Xavier S Chen J Lin CH Ratliff B Rabadi M. Endothelial sirtuin 1 deficiency perpetrates nephrosclerosis through downregulation of matrix metalloproteinase-14: relevance to fibrosis of vascular senescence. J Am Soc Nephrol (2014) 25(2):276–91. doi: 10.1681/ASN.2013010069 PMC3904558 24136919

[26] Haigis MC Guarente LP. Mammalian sirtuins–emerging roles in physiology, aging, and calorie restriction. Genes Dev (2006) 20(21):2913–21. doi: 10.1101/gad.1467506 17079682

[27] Potente M Ghaeni L Baldessari D Mostoslavsky R Rossig L Dequiedt F. SIRT1 controls endothelial angiogenic functions during vascular growth. Genes Dev (2007) 21(20):2644–58. doi: 10.1101/gad.435107 PMC2000327 17938244

[28] Ong ALC Ramasamy TS. Role of Sirtuin1-p53 regulatory axis in aging, cancer and cellular reprogramming. Ageing Res Rev (2018) 43:64–80. doi: 10.1016/j.arr.2018.02.004 29476819

[29] Kobayashi Y Furukawa-Hibi Y Chen C Horio Y Isobe K Ikeda K. SIRT1 is critical regulator of FOXO-mediated transcription in response to oxidative stress. Int J Mol Med (2005) 16(2):237–43. doi: 10.3892/ijmm.16.2.237 16012755

[30] Higashida K Kim SH Jung SR Asaka M Holloszy JO Han DH. Effects of resveratrol and SIRT1 on PGC-1alpha activity and mitochondrial biogenesis: a reevaluation. PloS Biol (2013) 11(7):e1001603. 23874150 10.1371/journal.pbio.1001603 PMC3706311

[31] de Mingo A de Gregorio E Moles A Tarrats N Tutusaus A Colell A. Cysteine cathepsins control hepatic NF-kappaB-dependent inflammation *via* sirtuin-1 regulation. Cell Death Dis (2016) 7(11):e2464. 27831566 10.1038/cddis.2016.368 PMC5260902

[32] Finkel T Deng CX Mostoslavsky R. Recent progress in the biology and physiology of sirtuins. Nature (2009) 460(7255):587–91. doi: 10.1038/nature08197 PMC3727385 19641587

[33] He G Karin M. NF-kappaB and STAT3 - key players in liver inflammation and cancer. Cell Res (2011) 21(1):159–68. doi: 10.1038/cr.2010.183 PMC3193410 21187858

[34] Liu T Zhang L Joo D Sun SC. NF-kappaB signaling in inflammation. Signal Transduct Target Ther (2017) 2. doi: 10.1038/sigtrans.2017.23 PMC5661633 29158945

[35] Qiu Y Zhou X Liu Y Tan S Li Y. The role of sirtuin-1 in immune response and systemic lupus erythematosus. Front Immunol (2021) 12:632383. doi: 10.3389/fimmu.2021.632383 33981300 PMC8110204

[36] Wang ZL Luo XF Li MT Xu D Zhou S Chen HZ. Resveratrol possesses protective effects in a pristane-induced lupus mouse model. PloS One (2014) 9(12):e114792. doi: 10.1371/journal.pone.0114792 25501752 PMC4263676

[37] Singh V Ubaid S. Role of silent information regulator 1 (SIRT1) in regulating oxidative stress and inflammation. Inflammation (2020) 43(5):1589–98. doi: 10.1007/s10753-020-01242-9 32410071

[38] Yang Y Liu Y Wang Y Chao Y Zhang J Jia Y. Regulation of SIRT1 and its roles in inflammation. Front Immunol (2022) 13:831168. doi: 10.3389/fimmu.2022.831168 35359990 PMC8962665

[39] Granchi C Minutolo F. Activators of sirtuin-1 and their involvement in cardioprotection. Curr Med Chem (2018) 25(34):4432–56. doi: 10.2174/0929867325666180214115438 29446717

[40] Sharma A Singh K Almasan A. Histone H2AX phosphorylation: a marker for DNA damage. Methods Mol Biol (2012) 920:613–26. doi: 10.1007/978-1-61779-998-3_40 22941631

[41] Plappert-Helbig U Libertini S Frieauff W Theil D Martus HJ. Gamma-H2AX immunofluorescence for the detection of tissue-specific genotoxicity *in vivo* . Environ Mol Mutagen (2019) 60(1):4–16. doi: 10.1002/em.22238 30307065

[42] Yang Y Luo S Huang J Xiao Y Fu Y Liu W. Photoactivation of innate immunity receptor TLR8 in live mammalian cells by genetic encoding of photocaged tyrosine. Chembiochem (2022) 23(4):e202100344. doi: 10.1002/cbic.202100344 34460982

[43] Gano LB Donato AJ Pasha HM Hearon CM Jr. Sindler AL Seals DR. The SIRT1 activator SRT1720 reverses vascular endothelial dysfunction, excessive superoxide production, and inflammation with aging in mice. Am J Physiol Heart Circ Physiol (2014) 307(12):H1754–63. doi: 10.1152/ajpheart.00377.2014 PMC4269699 25326534

[44] Thery C Amigorena S Raposo G Clayton A. Isolation and characterization of exosomes from cell culture supernatants and biological fluids. Curr Protoc Cell Biol (2006) Chapter 3:Unit 3 22. doi: 10.1002/0471143030.cb0322s30 18228490

[45] Thery C Witwer KW Aikawa E Alcaraz MJ Anderson JD Andriantsitohaina R. Minimal information for studies of extracellular vesicles 2018 (MISEV2018): a position statement of the international society for extracellular vesicles and update of the MISEV2014 guidelines. J Extracell Vesicles (2018) 7(1):1535750. doi: 10.1080/20013078.2018.1535750 30637094 PMC6322352

[46] Dragovic RA Gardiner C Brooks AS Tannetta DS Ferguson DJ Hole P. Sizing and phenotyping of cellular vesicles using nanoparticle tracking analysis. Nanomedicine (2011) 7(6):780–8. doi: 10.1016/j.nano.2011.04.003 PMC3280380 21601655

[47] Gardiner C Ferreira YJ Dragovic RA Redman CW Sargent IL. Extracellular vesicle sizing and enumeration by nanoparticle tracking analysis. J Extracell Vesicles (2013) 2. doi: 10.3402/jev.v2i0.19671 PMC3760643 24009893

[48] Tyanova S Temu T Sinitcyn P Carlson A Hein MY Geiger T. The Perseus computational platform for comprehensive analysis of (prote)omics data. Nat Methods (2016) 13(9):731–40. doi: 10.1038/nmeth.3901 27348712

[49] Teng F Fussenegger M. Shedding light on extracellular vesicle biogenesis and bioengineering. Adv Sci (Weinh) (2020) 8(1):2003505. 33437589 10.1002/advs.202003505 PMC7788585

[50] Borghesan M Fafian-Labora J Eleftheriadou O Carpintero-Fernandez P Paez-Ribes M Vizcay-Barrena G. Small extracellular vesicles are key regulators of non-cell autonomous intercellular communication in senescence *via* the interferon protein IFITM3. Cell Rep (2019) 27(13):3956–71 e6. doi: 10.1016/j.celrep.2019.05.095 31242426 PMC6613042

[51] Zhou Y Zhou B Pache L Chang M Khodabakhshi AH Tanaseichuk O. Metascape provides a biologist-oriented resource for the analysis of systems-level datasets. Nat Commun (2019) 10(1):1523. doi: 10.1038/s41467-019-09234-6 30944313 PMC6447622

[52] Yeung F Hoberg JE Ramsey CS Keller MD Jones DR Frye RA. Modulation of NF-kappaB-dependent transcription and cell survival by the SIRT1 deacetylase. EMBO J (2004) 23(12):2369–80. doi: 10.1038/sj.emboj.7600244 PMC423286 15152190

[53] Ota Y Takahashi K Otake S Tamaki Y Okada M Aso K. Extracellular vesicle-encapsulated miR-30e suppresses cholangiocarcinoma cell invasion and migration *via* inhibiting epithelial-mesenchymal transition. Oncotarget (2018) 9(23):16400–17. doi: 10.18632/oncotarget.24711 PMC5893249 29662654

[54] Chen K Wang Q Liu X Wang F Yang Y Tian X. Hypoxic pancreatic cancer derived exosomal miR-30b-5p promotes tumor angiogenesis by inhibiting GJA1 expression. Int J Biol Sci (2022) 18(3):1220–37. doi: 10.7150/ijbs.67675 PMC8771853 35173549

[55] Zang J Maxwell AP Simpson DA McKay GJ. Differential expression of urinary exosomal MicroRNAs miR-21-5p and miR-30b-5p in individuals with diabetic kidney disease. Sci Rep (2019) 9(1):10900. doi: 10.1038/s41598-019-47504-x 31358876 PMC6662907

[56] Klein JD Wang XH. Electrically stimulated acupuncture increases renal blood flow through exosome-carried miR-181. Am J Physiol Renal Physiol (2018) 315(6):F1542–F9. doi: 10.1152/ajprenal.00259.2018 PMC6336992 30132347

[57] Groot M Lee H. Sorting mechanisms for MicroRNAs into extracellular vesicles and their associated diseases. Cells (2020) 9(4). doi: 10.3390/cells9041044 PMC7226101 32331346

[58] Fabbiano F Corsi J Gurrieri E Trevisan C Notarangelo M D'Agostino VG. RNA Packaging into extracellular vesicles: An orchestra of RNA-binding proteins? J Extracell Vesicles (2020) 10(2):e12043. doi: 10.1002/jev2.12043 33391635 PMC7769857

[59] Mensa E Guescini M Giuliani A Bacalini MG Ramini D Corleone G. Small extracellular vesicles deliver miR-21 and miR-217 as pro-senescence effectors to endothelial cells. J Extracell Vesicles (2020) 9(1):1725285. doi: 10.1080/20013078.2020.1725285 32158519 PMC7048230

[60] Zhang H Freitas D Kim HS Fabijanic K Li Z Chen H. Identification of distinct nanoparticles and subsets of extracellular vesicles by asymmetric flow field-flow fractionation. Nat Cell Biol (2018) 20(3):332–43. doi: 10.1038/s41556-018-0040-4 PMC5931706 29459780

[61] Terlecki-Zaniewicz L Pils V Bobbili MR Lammermann I Perrotta I Grillenberger T. Extracellular vesicles in human skin: Cross-talk from senescent fibroblasts to keratinocytes by miRNAs. J Invest Dermatol (2019) 139(12):2425–+. doi: 10.1016/j.jid.2019.05.015 31220456

[62] Lehmann BD Paine MS Brooks AM McCubrey JA Renegar RH Wang R. Senescence-associated exosome release from human prostate cancer cells. Cancer Res (2008) 68(19):7864–71. doi: 10.1158/0008-5472.CAN-07-6538 PMC3845029 18829542

[63] Dellago H Preschitz-Kammerhofer B Terlecki-Zaniewicz L Schreiner C Fortschegger K Chang MW. High levels of oncomiR-21 contribute to the senescence-induced growth arrest in normal human cells and its knock-down increases the replicative lifespan. Aging Cell (2013) 12(3):446–58. doi: 10.1111/acel.12069 PMC3864473 23496142

[64] Zhang G Esteve PO Chin HG Terragni J Dai N Correa IR Jr.. Small RNA-mediated DNA (cytosine-5) methyltransferase 1 inhibition leads to aberrant DNA methylation. Nucleic Acids Res (2015) 43(12):6112–24. doi: 10.1093/nar/gkv518 PMC4499142 25990724

