# Supplementary material for: Extracellular vesicle-associated microRNA-30b-5p activates macrophages through the SIRT1/ NF-κB pathway in cell senescence
Source: Front Immunol. 2022 Aug 31;13:955175. doi: 10.3389/fimmu.2022.955175 (PMC9471260; doi:10.3389/fimmu.2022.955175)
Supplement: Supplementary file 1 [file DataSheet_1.pdf]

Supplementary Materials for

Extracellular vesicle-associated microRNA-30b-5p activates macrophages through the SIRT1/ NF-κB pathway in cell senescence

Yu Xiao[1], Jiaqi Liang[2,3,4], Kenneth W. Witwer[5,6], Ying Zhang[2,3,4], Qian Wang[1*], Hang Yin[2,3,4*]

[*]Correspondence:

H. Yin (yin_hang@tsinghua.edu.cn);

Q. Wang (wangqian@smu.edu.cn)

This file includes:

Figures S1 to S4
Tables S1 to S2

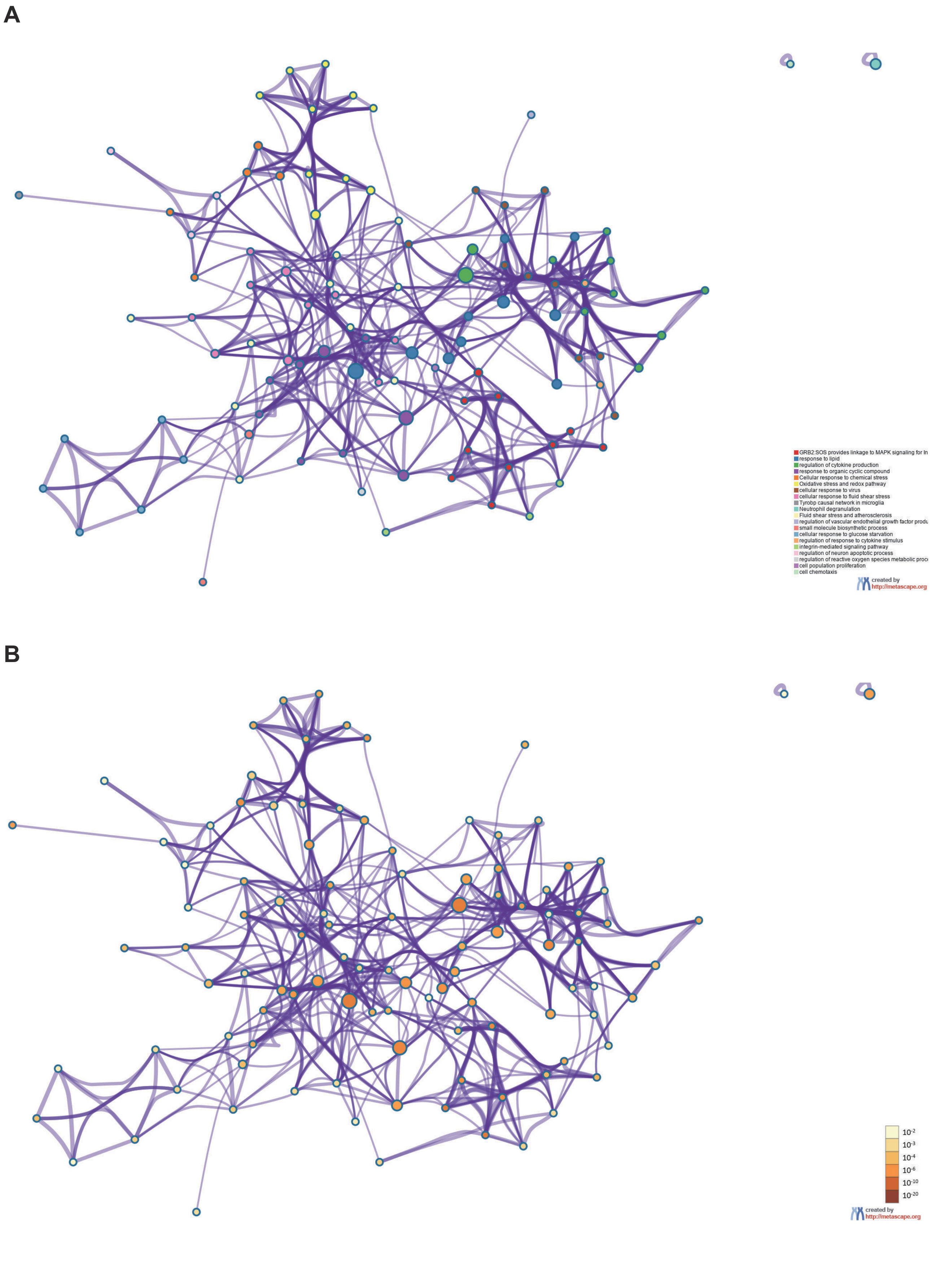
A
GRB2:SOS provides linkage to MAPK signaling for In
response to lipid
regulation of cytokine production
response to organic cyclic compound
Cellular response to chemical stress
Oxidative stress and redox pathway
cellular response to virus
cellular response to fluid shear stress
Tyrobp causal network in microglia
Neutrophil degranulation
Fluid shear stress and atherosclerosis
regulation of vascular endothelial growth factor produ
small molecule biosynthetic process
cellular response to glucose starvation
regulation of response to cytokine stimulus
integrin-mediated signaling pathway
regulation of neuron apoptotic process
regulation of reactive oxygen species metabolic proc
cell population proliferation
cell chemotaxis
created by http://metascape.org
B
10^{-2}
10^{-3}
10^{-4}
10^{-6}
10^{-10}
10^{-20}
created by http://metascape.org

C

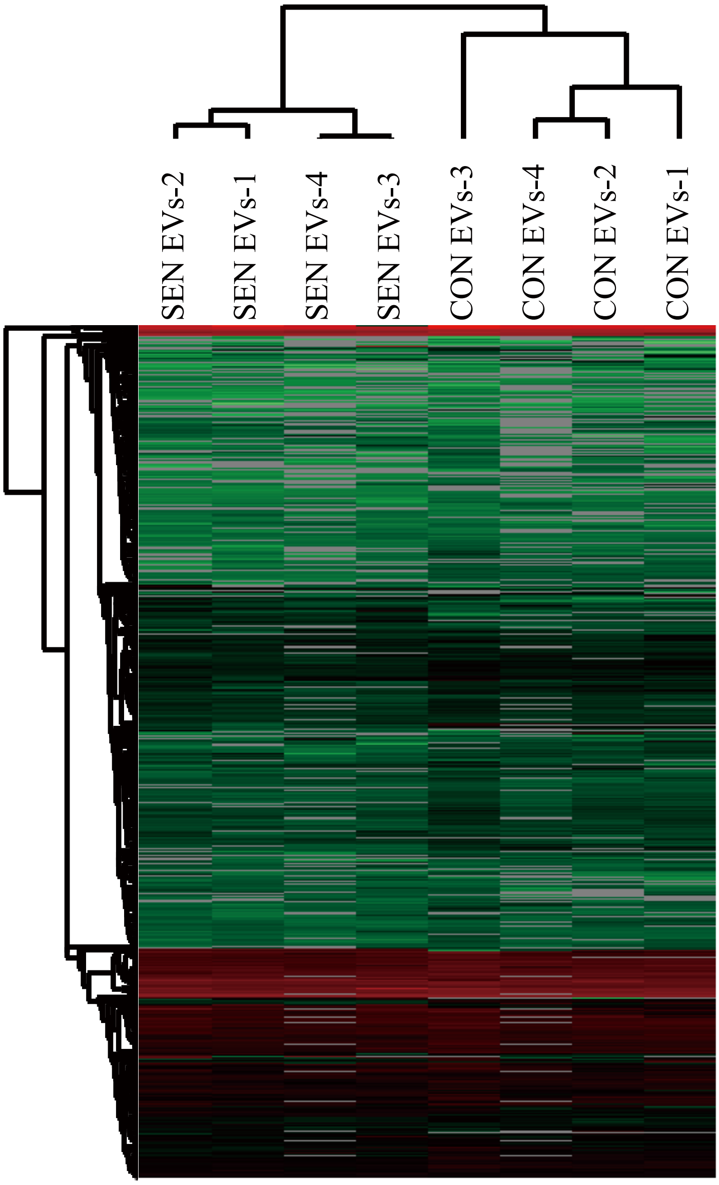
SEN EVs-2
SEN EVs-1
SEN EVs-4
SEN EVs-3
CON EVs-3
CON EVs-4
CON EVs-2
CON EVs-1

D

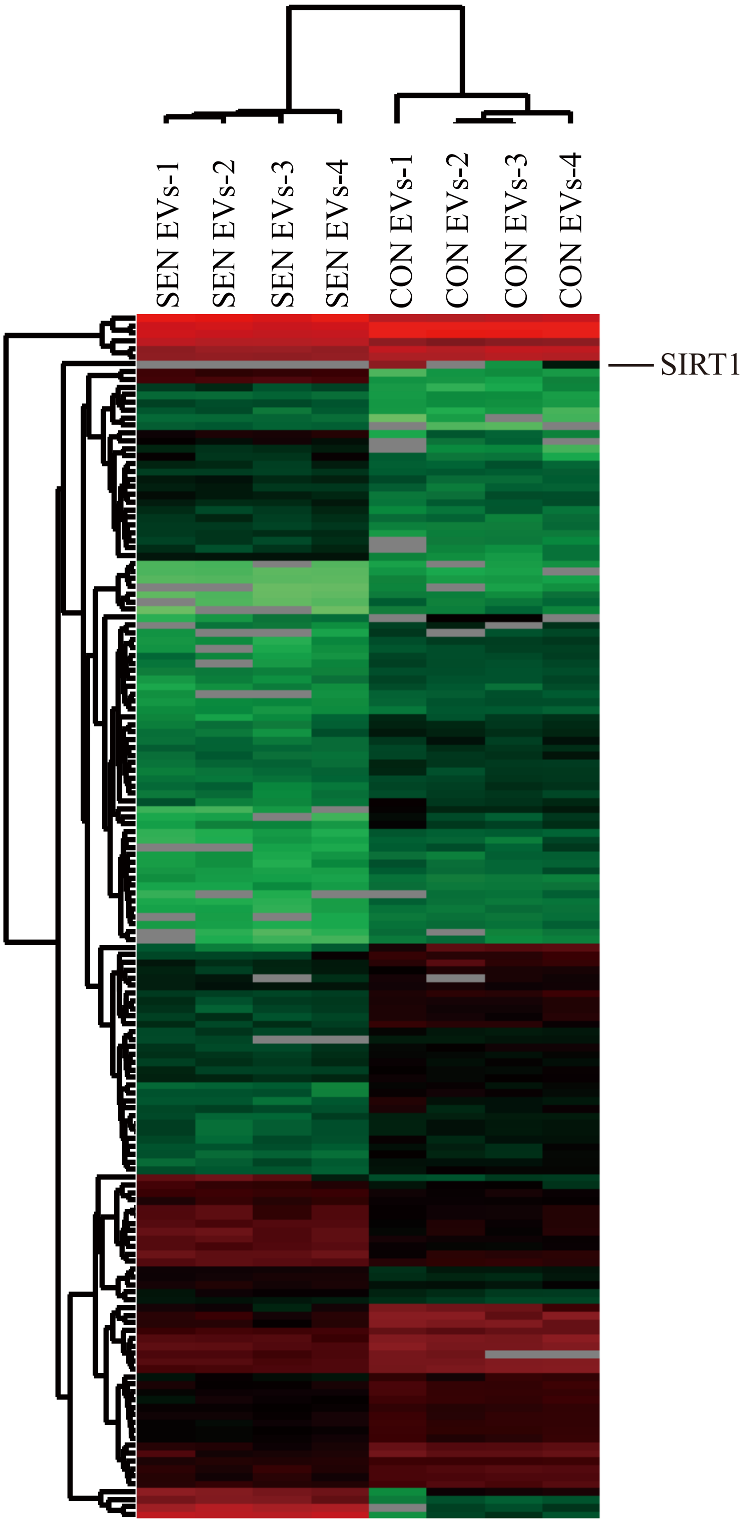
SEN EVs-1
SEN EVs-2
SEN EVs-3
SEN EVs-4
CON EVs-1
CON EVs-2
CON EVs-3
CON EVs-4
SIRT1

Figure S1. Network of enriched terms in proteomic study and heatmaps: (A) Colored by cluster ID, where nodes that share the same cluster ID are typically close to each other; (B) Colored by *p*-value, where terms containing more genes tend to have a more significant *p*-value; (C) Heatmap of identified proteinsfrom LC-MS/MS; (D) Heatmap of differential proteins.

A

mmu-miR-30b-5p

```
Target:  5' uagcaugucaaaaaaUGAAUGUu
                           | | | | | | |
miRNA: 3' ugaguggcugucgcaACUUACAa
```

Position 72-78 of Sirt1 3'UTR

Site type	Context++ score	Context++ score percentile	Weighten context++ score	Conserved branch length	P_{CT}
7mer-m8	-0.21	85	-0.21	3.891	0.65

Figure S2. (A) The TargetScan and ENCORI prediction tool was used to predict the target of miR-30b-5p

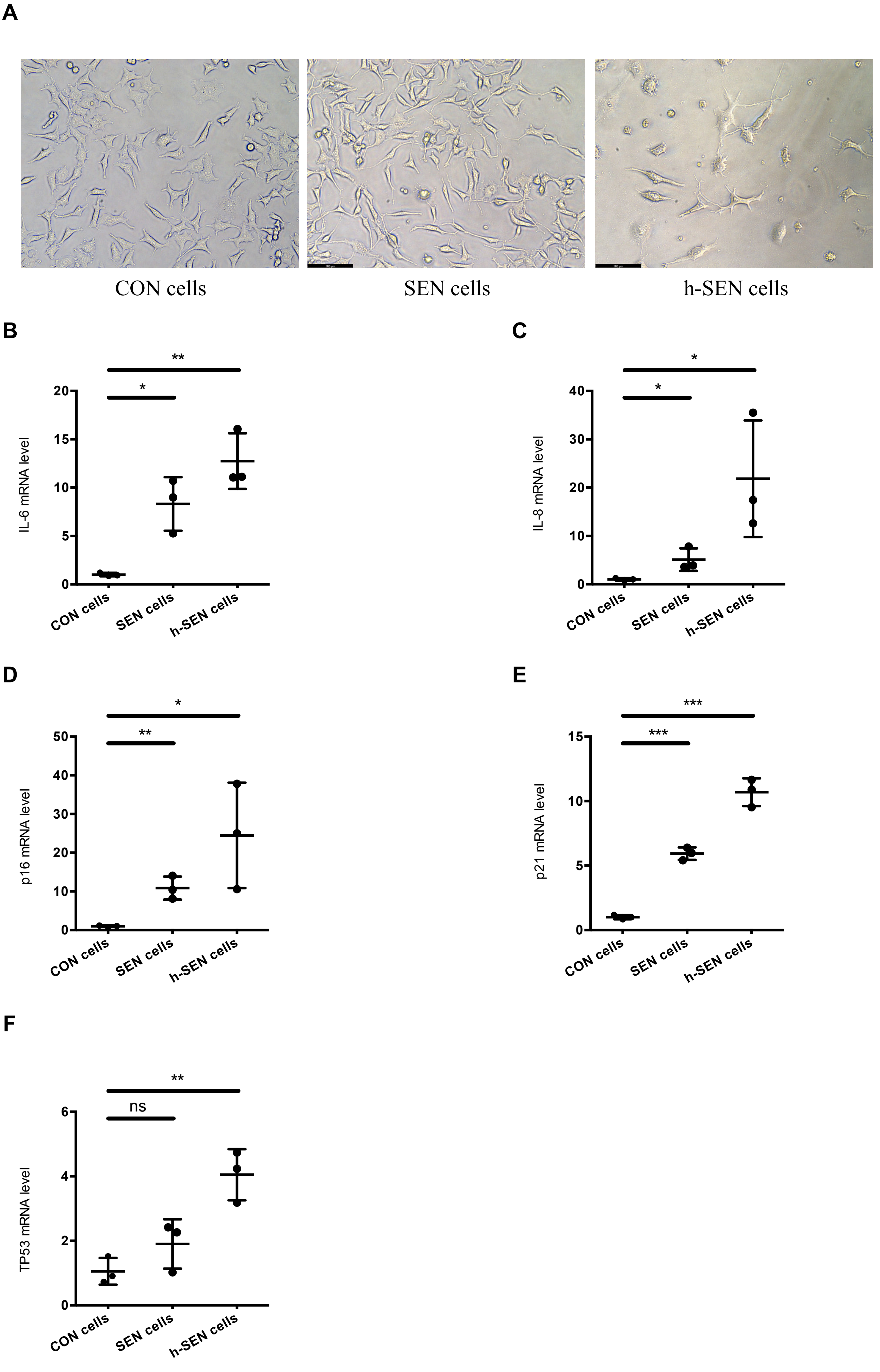
A
CON cells
SEN cells
h-SEN cells
B
IL-6 mRNA level
20
15
10
5
0
**
*
CON cells
SEN cells
h-SEN cells
C
IL-8 mRNA level
40
30
20
10
0
*
*
CON cells
SEN cells
h-SEN cells
D
p16 mRNA level
50
40
30
20
10
0
*
**
CON cells
SEN cells
h-SEN cells
E
p21 mRNA level
15
10
5
0
***
***
CON cells
SEN cells
h-SEN cells
F
TP53 mRNA level
6
4
2
0
**
ns
CON cells
SEN cells
h-SEN cells

Figure S3. (A) The morphology of CON, SEN, and h-SEN cells; (B-F) mRNA levels of senescence markers of CON, SEN, and h-SEN cells.

A

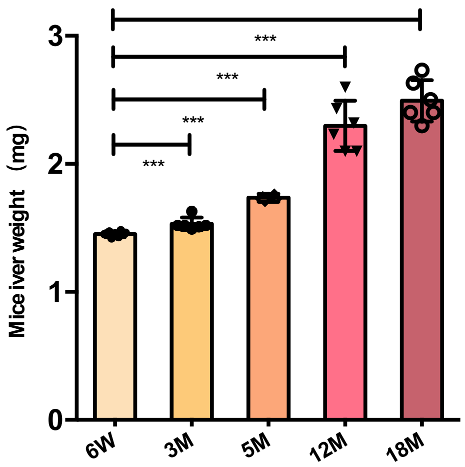

Figure S4. (A) Liver weights of mice at different ages.

TABLE S1 The reagents used in the study

REAGENT or RESOURCE	SOURCE	IDENTIFIER
CD63 antibody	Abcam	ab59479
Calnexin antibody	Abcam	ab133615
ALIX antibody	Proteintech	12422-1-AP
phospho-Histone H2A.X (Ser139) (20E3)	Cell Signaling Technology	#9718
SIRT1 (D1D7) Rabbit mAb	Cell Signaling Technology	#9475
β-actin (13E5) Rabbit mAb	Cell Signaling Technology	#4970
NF-κB p65 antibody	Proteintech	80979-1-RR
Alexa Fluor 488 goat anti-rabbit IgG (H+L)	Invitrogen	A11034
SIRT1 agonist SRT1720	MedChemExpress	HY-10532
miR-30b-5p antagonist	GenePharma	/

TABLE S2 The up-regulated protein in proteomics study

Uniport accession	log2(Student's T-test Difference senescence EV_ctrl EV)	N: -Log Student's T-test p-value senescence EV_ctrl EV
A0A0R4J027	5.70265	4.868
Q05769	4.84491	6.02155
A0A0R4J2B2	4.45789	6.97709
Q64339	4.4276	5.03593
Q64337	4.32737	5.54507
P10923	3.0221	1.98663
P14901	2.98244	3.538
Q8R5A3	2.76541	2.19345
Q9JIA7	2.50324	5.1709
P0DOV2	2.41604	2.91156
A0A494B9Y5	2.36364	4.91459
P28667	2.08779	2.84957
P05480	2.0241	3.35682
Q8CBA2	2.00871	2.14696
Q05816	2.00186	2.2665
Q8R4B8	1.71062	4.14025
P10852	1.65825	4.93537
Q9D1A2	1.6335	2.9248
Q8K363	1.62421	2.79094
D3Z2E7	1.58876	2.62023
Q8BTY2	1.5337	5.18448
Q61024	1.51325	5.14708
O55098	1.41028	2.75459
Q9JHK5	1.40038	3.37517
P30993	1.37922	3.3047
Q8BH04	1.33764	3.25911
Q9JM90	1.33367	2.49144
P99029	1.30423	3.1962
Q6Q899	1.26538	4.06699
Q60766	1.26411	2.44973
Q8BV66	1.25224	2.81662
Q9WUM3	1.23185	3.32039
Q99JI6	1.17999	2.62107
E9Q555	1.1794	2.87061
P62835	1.17748	2.61978
P17809	1.14157	4.25912
Q9BDB7	1.07761	3.00484
O08807	1.00818	4.55779
Q923D2	1.00517	2.76608
Q8K2Q7	0.923204	3.79569

P24288	0.904095	3.22316
P26638	0.883189	4.71491
Q8BGQ7	0.87041	4.44913
P35700	0.86259	3.2988
O70145	0.801276	3.91453
G3XA17	0.766078	3.07411
P37040	0.70355	3.10144
P25799	0.623432	4.22342
